# Designing an operational research TB training program in Zambia

**DOI:** 10.5588/pha.23.0046

**Published:** 2024-03-01

**Authors:** R. Kumar, J. Bwembya, V. Makwambeni, V. Musonda, R. Chimzizi, A. Mwinga

**Affiliations:** ^1^ United States Agency for International Development (USAID) Eradicate TB Project, Lusaka,; ^2^ ZAMBART, Lusaka,; ^3^ PATH, Lusaka,; ^4^ USAID Sustaining Technical and Analytical Resources (STAR) Project, Ministry of Health, National TB and Leprosy Control Program, Lusaka, Zambia

**Keywords:** tuberculosis, Zambia, OR, capacity strengthening, healthcare workers, Africa

## Abstract

**BACKGROUND:**

The USAID-funded Eradicate TB Project (ETB) partnered with the National Tuberculosis and Leprosy Control Program (NTLP) to establish an operational research (OR) training program in order to generate local evidence to enhance TB care in Zambia.

**METHOD:**

Between 2017 and 2021, healthcare workers (HCWs) from district teams underwent two 10-day intensive training sessions. The program evolved to include a competitive application process and an additional primer workshop on developing feasible research questions.

**RESULTS:**

Of the 36 enrollees in the OR training program, 26 (72.2%) completed it, leading to nine OR studies that informed interventions for TB care improvement. Notable achievements include reduced TB mortality, increased pediatric notifications, and enhanced sputum courier systems, with all studies disseminated at national and international conferences, four submitted to peer-reviewed journals, of which three were published. Two studies were replicated by the NTLP at provincial and national levels.

**CONCLUSIONS:**

Integrating OR training into TB initiatives is feasible and beneficial. The program's phased execution and adaptive strategies provide valuable insights for similar settings, although challenges in sustainability of mentorship and funding persist. This success underscores the importance of continuous OR capacity strengthening among HCWs in Zambia.

## Abstract (fr)

**CONTEXTE:**

Le projet Eradicate TB (ETB) financé par l'USAID s'est associé au programme National de contrôle de la Tuberculose et de la Lèpre (NTLP) pour mettre en place un programme de formation à la recherche opérationnelle (OR), afin de générer des preuves locales pour améliorer les soins de la TB en Zambie.

**MÉTHODE:**

Entre 2017 et 2021, des travailleurs de la santé (HCWs) provenant d'équipes de district ont suivi deux sessions de formation intensive de 10 jours. Le programme a évolué pour inclure un processus de candidature concurrentiel et un atelier d'initiation supplémentaire sur l'élaboration de questions de recherche réalisables.

**RÉSULTATS:**

Vingt-six (72,2%) des HCW inscrits au programme ont terminé leur formation, ce qui a donné lieu à neuf études de OR qui ont servi de base à des interventions visant à améliorer les soins antituberculeux. Parmi les réalisations notables, citons la réduction de la mortalité due à la TB, l'augmentation des notifications pédiatriques et l'amélioration des systèmes de messagerie des expectorations. Toutes les études ont été diffusées lors de conférences nationales et internationales, quatre ont été soumises à des revues à comité de lecture et trois ont été publiées. Deux études ont été reproduites par le NTLP aux niveaux provincial et national.

**CONCLUSIONS:**

L'intégration de la formation OR dans les initiatives de lutte contre la TB est faisable et bénéfique. L'exécution progressive du programme et les stratégies adaptatives fournissent des indications précieuses pour des contextes similaires, bien que les défis liés à la durabilité du mentorat et du financement persistent. Ce succès souligne l'importance d'un renforcement continu des capacités OR parmi les HCW en Zambie.

Universal health coverage and healthcare excellence necessitate locally generated evidence. Operational research (OR) is a cost-effective method to enhance health services by investigating and solving health system problems. Embedding OR into disease programs improves health system performance, tests new interventions, and informs policy changes.^1^

In low and middle-income countries such as Zambia, OR capacity is limited, especially at decentralized levels. Initiatives such as the WHO-TDR's Structured Operational Research and Training Initiative (SORT IT) provide practical OR training, have been adapted across Africa to tackle local health challenges, including TB and neglected diseases, and post-outbreak health system recovery.^2–4^

The USAID-funded Eradicate TB Project (ETB) in collaboration with the Zambia National TB and Leprosy Program (NTLP), as well as local partners, including PATH Zambia (Lusaka, Zambia) and ZAMBART (Lusaka, Zambia), aimed to strengthen capacity of healthcare workers (HCWs) to conduct OR, focusing on TB care and control. The ETB OR training program aimed to enhance the capacity of district-level Ministry of Health HCWs to identify service gaps, conduct relevant research, and develop low-cost solutions for better TB care and control. This paper details the iterative design and execution of the ETB's OR training program, providing insights into OR implementation in health programs.

## METHODS

A research capacity strengthening program modeled after SORT IT involved two cohorts (2017–2019, 2019–2021). Success was evaluated based on completed studies, presentations, and publications.

A survey administered 12 months post-training captured trainees' assessment of their personal improvement in OR application, the impact of their OR study on TB program performance, the broader application of learned skills, and the ability to mentor or support colleagues. Responses from ordinal questions were analyzed using MS Excel v16 (Microsoft, Redmond, WA, USA) to generate frequencies and percentages. To complement our survey findings, we conducted thematic analysis of participants' qualitative responses. This involved generating codes, identifying and reviewing themes, and defining them in relation to our survey results. Selected quotes illustrating these themes were used to enrich our understanding of the quantitative data.

### Implementing the original program

In 2017, the NTLP selected four districts for improved TB service delivery based on high TB notification rates, high-risk sub-populations, and HCWs with some research skills. Eighteen HCWs from four district teams, including TB focal persons, laboratory staff, and health directors, underwent a year-long training. This involved two 10-day intensive sessions where teams developed OR proposals, analyzed data, wrote reports, and designed short-term interventions.

### Redesigning the program

Through several key stakeholder meetings, we identified challenges that led to a redesign of the training approach. During the trainee selection process, priority was initially given to roles in the TB program over research interests or motivation, leading to a lack of engagement and significant delays in producing manuscripts of publishable quality.

We transitioned to a competitive application process to attract individuals interested in OR, detailed in the Supplementary Data. This change, along with a new 3-day workshop for applicants to pitch their research questions to the NTLP, was designed to boost commitment, enhance research quality, and ensure dedication to service improvement.

We also addressed inherent hierarchies within district teams that affected collaborative research efforts, by focusing training on a primary investigator and district TB focal person to lead research within their teams. To expedite the review process, we improved coordination with partners for quicker ethics approval and introduced flexible mentorship, blending in-person and virtual support, to ensure consistent guidance and trainee development.

### Implementing the redesigned program

The redesigned OR program's second cohort drew over 100 applicants. Applicants were scored based on their previous research experience, familiarity with routine programmatic TB data, and personal motivations for promoting and building capacity in OR. The top ranked candidates participated in a 3-day primer workshop to develop research questions aligned with Zambia's TB priorities, culminating in presentations to NTLP and ETB representatives who selected the top research topics.

Selected investigators then participated in a 6-day proposal development workshop, refining their proposals with daily facilitator feedback. Non-TB focal principal investigators were paired with TB focal co-investigators. From October to December 2019, five proposals were approved following thorough reviews. Trainees subsequently collected study data from January to June 2020, leading to a final 6-day workshop on data analysis and manuscript preparation.

### Course costs and funding

Funded by USAID, the program had an annual OR training and mentorship budget ranging from US$27,000 to US$40,000. Median costs were US$47,170 for training delivery and mentorship, US$908 per trainee, and US$2,708 per research project. District health offices additionally supported data collection and dissemination efforts.

## RESULTS

Of the 36 enrollees in the OR training program, 26 (72.2%) completed it, implementing nine OR studies across diverse topics from identifying reasons for losses in the sputum sample referral cascade in rural districts;^5,6^ to factors affecting TB patient mortality (Table 1).^7^ The solutions devised by these teams, such as district-wide training in data quality and diagnostic aids, were cost-effective and mitigated local service gaps. All nine study abstracts were presented at the 2019 and 2020 Union Conference on Lung Health and the Zambia National Health Research Authority’s Annual Conference. Furthermore, four studies were submitted to peer-reviewed journals, and three were published.

**Table 1.** Overview of operational research contributions and dissemination in Zambia's TB control program, 2017–2021.

District	Study title	Presentation forum	Publication progress
Cohort 1			
Chingola	Identification of risk factors for unfavorable TB treatment outcomes among patients in Chingola, Zambia	Abstract presented at Union Conference 2019	Not published
Kapiri Mposhi	Adult TB care reporting cascade in 4 TB diagnostic centers in Kapiri Mposhi, Zambia	Abstract presented at Union Conference 2019	Not published
Mpulungu	Investigation of staff and non-staff-related factors contributing to low sputum sample referrals in Mpulungu, Zambia	Abstract presented at Union Conference 2019	Published^5^
Ndola	Investigating leakages in the pediatric TB care cascade through examination of patient records from point of entry to the point of TB notification: a case study of Ndola District	Abstract presented at Union Conference 2019	Not published
Cohort 2			
Chililabombwe	An assessment of the extent to which follow-up sputum smear microscopy is performed for drug-susceptible pulmonary tuberculosis patients in Chililabombwe, Zambia	Abstract presented at Zambia National Health Research Conference 2020	Not published
Itezhi-Tezhi	Mortality and associated risk factors among TB patients in Itezhi Tezhi, Zambia	Abstract presented at Union Conference 2020	Published^7^
Mansa	GeneXpert MTB/RIF under-utilization: knowledge, attitude, and practices of health care workers in Samfya, Zambia	Abstract presented at Zambia National Health Research Conference 2020	Not published
Mpongwe	Investigation of losses along the sputum sample referral system in rural Mpongwe District, Copperbelt, Zambia	Abstract presented at Zambia National Health Research Conference 2020	Published^6^
Kitwe	Factors contributing to low childhood TB case notification in Kitwe, Zambia	Abstract presented at Zambia National Health Research Conference 2020	Submitted to journal

The program's impact extended beyond initial studies, prompting larger-scale research influenced by the original work. For example, an analysis of pediatric TB in Ndola led to a broader city-wide diagnostic intervention, while a rural study on TB mortality^7^ catalyzed a comprehensive national mortality study. Trainees' expertise was pivotal in scaling up these interventions with their involvement in study design and implementation at provincial and national levels.

In a self-assessment survey of 15 (42%) program graduates (Table 2), participants reported significant improvements in their ability to apply OR methodologies to identify and address gaps in health service delivery in a cost-effective way. One program graduate described:

**Table 2.** Self-assessment of OR training outcomes by program graduates (*n* = 15).

		Strongly disagree	Disagree	Neutral	Agree	Strongly agree
Training outcomes	Statement	*n* (%)	*n* (%)	*n* (%)	*n* (%)	*n* (%)
Self-reported improvement in OR skill application	My ability to utilize OR methodologies to identify and/or close gaps in the delivery of health services has improved as a result of my attending this training	2 (13.3)	0	0	5 (33.3)	8 (53.3)
Utilization of OR skills in TB program improvement	I have been able to utilize OR skills to identify and/or close gaps in the TB program for my province/district/facility	2 (13.3)	0	0	8 (53.3)	5 (33.3)
Impact of OR study on TB program performance	The OR study implemented as part of the training has helped improve performance of the TB program in my province/district/health facility	2 (13.3)	0	0	8 (53.3)	5 (33.3)
Application of OR skills beyond TB program	I have been able to apply the skills I acquired from this training to conduct research in health services other than the TB program	1 (6.7)	1 (6.7)	1 (6.7)	8 (53.3)	4 (26.7)
Peer support and research facilitation post-training	I have been able to support my co-workers to conduct research following the ETB OR training I attended	1 (6.7)	1 (6.7)	2 (13.3)	6 (40.0)	5 (33.3)

OR = operational research.

[The training program] helped me understand that l can do research with minimum, or with no funds.

A majority (87%) indicated that their skills in utilizing OR within the TB program had been improved, directly contributing to program improvements in their respective districts. As a program graduate observed:

All [suspected TB patients] are well-documented, and follow-up [has been] made easier. Further, the samples are not getting lost in the sputum sample referral system.

The majority (87%) agreed that implementing OR studies as part of their training had improved the performance of their district’s TB program, with tangible impacts observed at the health facility level. Beyond TB-focused research, graduates (73%) also reported successfully applying their acquired OR skills to other health services, suggesting a transferable benefit of the training. A program graduate noted:

Currently, I'm writing two scientific articles [about] my province's experiences with the COVID-19 pandemic, and predictors of COVID-19 outcomes in hospitalized patients at Mansa General Hospital [Mansa, Zambia]. I am applying some of the skills I learn[ed] from [the] OR training.

Most graduates (73%) also assisted colleagues to conduct research after training, thereby fostering a collaborative and continuous learning culture within their work settings.

## DISCUSSION

The success of Zambia's OR program can be attributed to its practical, hands-on mentorship and 'learning-by-doing' approach, which included guidance on technical research aspects from proposal writing to data analysis. This model mirrors the successful strategies of the Rwanda OR program, which also reported high completion and publication rates, alongside notable skills development.^8^

One factor that hindered the first cohort from producing manuscripts of publishable quality was the scarcity of mentors, similar to challenges faced in the Rwanda program. Zambia responded by leveraging local experts from the University of Zambia School of Public Health (Lusaka, Zambia) to minimize costs. However, for future programs, we recommend developing junior mentors within the program and utilizing blended learning methods.^9^ These strategies have successfully addressed mentorship gaps in Zambia's capacity-strengthening initiatives, and can ensure sustained mentorship to enhance the quality of research output.^10^

While the direct effectiveness of health service improvements by trainees remains to be evaluated with follow-up studies, the importance of dedicated funding for capacity strengthening, alongside OR, is clear for sustaining improved health service delivery. Current success metrics, predominantly based on journal publications and conference presentations, do not fully capture the program's impact.^3,11,12^ A broader measure of success should recognize the influence of trainee-led research on national studies and their active role as experts.

Looking ahead, capacity-building efforts must navigate the challenge of quantifying their impact on health policies and practices, as well as assessing the real-world value of the skills imparted to trainees.

## CONCLUSIONS

Our study findings suggest that integrating an OR training program into a donor project is feasible. The redesigning of the program is an example of how to adapt to unique national needs at the early stages of OR capacity strengthening when available mentorship is limited in-country. The study also demonstrates different iterative approaches to selecting trainees for these types of programs, such as a district-wide approach vs. individual, and best practices for engaging the NTLP to ensure buy-in and mentorship through the trainee process. The NTLP and future TB projects should build on our training program to further strengthen OR capacity among HCWs in Zambia.

## Supplementary Material



## References

[1] Zachariah R, . Nationalizing Operational Research Capacity Building: Necessity or Luxury? Ann Glob Health 2020;86(1):136. 33134092 10.5334/aogh.3056 PMC7583212

[2] Odhiambo J, . Adapting operational research training to the Rwandan context: the Intermediate Operational Research Training programme. Glob Health Action 2017;10(1):1386930. 29119872 10.1080/16549716.2017.1386930 PMC5700541

[3] Naidoo P, . How do we measure the success of operational research? Public Health Action 2014;4(2):1. 26393076 10.5588/pha.14.0056 PMC4539042

[4] Owiti P, . Structured operational research and training in the public health sector: The Kenyan experience. East Afr Med J 2016;93:S1–2.

[5] Goma R, . Losses in the sputum specimen referral cascade in Mpulungu District, Zambia: a cross-sectional study. Int J Environ Res Public Health 2022;19(3):1621. 35162643 10.3390/ijerph19031621 PMC8834727

[6] Nkhoma L, . Losses along the tuberculosis sputum sample referral cascade for Mpongwe District, Zambia. Afr J Prim Health Care Fam Med 2023;15(1):e1–7. 10.4102/phcfm.v15i1.3710 PMC9982457 36861920

[7] Chaaba E, . Mortality among persons receiving tuberculosis treatment in Itezhi-Tezhi District of Zambia: a retrospective cohort study. PLoS Glob Public Health 2023;3(2):e0001234. 36962999 10.1371/journal.pgph.0001234 PMC10021721

[8] Odhiambo J, . Implementation, outputs, and cost of a national operational research training in Rwanda. Ann Glob Health 2020;86(1):93. 32832387 10.5334/aogh.2933 PMC7413170

[9] Decroo T, . Blended SORT-IT for operational research capacity building: the model, its successes and challenges. Glob Health Action 2018;11(1):1469215. 29745782 10.1080/16549716.2018.1469215 PMC5954484

[10] Kumar R, . Zambia field epidemiology training program: strengthening health security through workforce development. Pan Afr Med J 2020;36:323. 33193977 10.11604/pamj.2020.36.323.20917 PMC7603807

[11] Feng N, . Operational research capacity building through the Structured Operational Research Training Initiative (SORT-IT) in China: implementation, outcomes and challenges. Infect Dis Poverty 2021;10(1):80. 34074332 10.1186/s40249-021-00865-w PMC8170796

[12] Zachariah R, . The published research paper: is it an important indicator of successful operational research at programme level? The published research paper. Trop Med Int Health 2010;15(11):1274–1277. 20976874 10.1111/j.1365-3156.2010.02630.x

