# Supplementary material for: Designing an operational research TB training program in Zambia
Source: Public Health Action. 2024 Mar 1;14(1):26–9. doi: 10.5588/pha.23.0046 (PMC11122706; doi:10.5588/pha.23.0046)
Supplement: Supplementary file 1 [file iutld_pha_23.0046_supplementarydata1.pdf]

http://dx.doi.org/10.5588/pha.23.0046

SUPPLEMENTARY DATA

Designing an operational research TB training program in Zambia

APPLICATION TO THE ZAMBIA OPERATIONAL RESEARCH TRAINING PROGRAM

The redesigned OR Training programs included baseline requirements, recommendations, and application questions which aimed to elicit reflection about current, pressing TB problems that they noticed in their districts, as well as TB Data sources available, and desire to make an impact in local TB service delivery.

REQUIREMENTS:

1. Working within the following provinces: North-Western, Copperbelt, Central, Luapula, Northern, or Muchinga.

2. Working in the District Health Office TB program in any role (ex: TB focal point, lab specialist, DHIO, surveillance officer).

3. Letter of Support from your supervisor for the full 9 month program if you are selected.

RECOMMENDED:

Motivated applicants with prior exposure to research (academic or applied) or quality improvement projects are encouraged to apply for this training.

APPLICATION QUESTIONS:

1. Which District Health Office do you currently work in?
2. What is your current position in the District Health Office?
3. What is your highest qualification?
4. What is your gender?
5. What is your age?

6. Give a detailed description of a TB-related problem that you have observed in your district or health facility that you would like to do research on. (Topics might include poor TB data capturing, low sputum sample referral, poor patient screening practices)

7. List some TB data sources that exist within your district that you might use to investigate the problem indicated in Q6. (examples: TB registers, facility TB reports)

8. Describe any research or Quality Improvement project that you have been involved in (does not need to be TB-specific).

9. Describe how you hope to use the skills you learn from this training to improving the TB program within your district.
